# N-glycosylation of ephrin B1 modulates its function and confers therapeutic potential in B-cell lymphoma

**DOI:** 10.1016/j.jbc.2025.108229

**Published:** 2025-01-27

**Authors:** Xiaoxi Li, Yong Jiang, Minyao Deng, Chenxiao Zhang, Hua Tang

**Affiliations:** 1 Department of Laboratory Medicine, School of Medicine, Jiangsu University, Zhenjiang, Jiangsu, China; 2 State Key Laboratory of Pharmaceutical Biotechnology, Department of Biochemistry, School of Life Sciences, Nanjing University, Nanjing, China

**Keywords:** N-linked glycosylation, anticancer drug, cancer therapy, lymphoma, stromal cell, tumor microenvironment, Eph–Ephrin system, ephrin B1, EFNB1-RBD-Fc

## Abstract

Given the pivotal role of the Eph–Ephrin signaling pathway in tumor progression, agonists or antagonists targeting Eph–Ephrin have emerged as promising anticancer strategies. However, the implications of glycosylation modifications within Eph–Ephrin and their targeted protein therapeutics remain elusive. Here, we identify that N-glycosylation within the receptor-binding domain (RBD) of ephrin B1 (EFNB1) is indispensable for its functional repertoire. Notably, compared with wildtype EFNB1, the glycosylation-deficient N139D mutant drastically diminishes the sensitivity of tumor cells with chemotherapeutic agents, suggesting the existence of both glycosylation-dependent and -independent effects mediated by EFNB1. Transcriptomic analysis highlights immune response and oxidative phosphorylation as the primary signaling pathways modulated by glycosylation modifications. In coculture systems, the EFNB1-RBD-Fc recombinant protein, while inhibiting B-lymphoma cells, also exerts differential impacts on stromal cells depending on their glycosylation status. Furthermore, the efficacy of both glycosylated and nonglycosylated EFNB1-RBD-Fc is influenced by the endogenous EFNB1 levels within tumor cells. Taking together, this study demonstrates the complexity and multifaceted roles of glycosylation in modulating EFNB1 function. These findings underscore the need for a nuanced understanding of glycosylation patterns in Eph–Ephrin-targeted therapies to optimize their therapeutic potential.

The Eph receptors and their Ephrin ligands play multifaceted roles in tumor progression, rendering them highly sought-after therapeutic targets. The Ephrin receptor family comprises a diverse array of receptors and ligands, specifically nine Eph-A receptors, five Eph-B receptors, along with five Ephrin-A ligands and three Ephrin-B ligands. This extensive repertoire underscores the complexity and versatility of the Ephrin signaling system. The canonical activation mechanism of Eph and Ephrin involves the mutual recognition and interaction between the ectodomains of Eph receptors and Ephrin ligands located in distinct cells, triggering bidirectional signaling within the corresponding cells (1, 2). Targeting the Eph–Ephrin interaction has emerged as a critical anticancer strategy, with a repertoire of therapeutic agents already in existence, including antibody-based antagonists, Fc fusion proteins that function as either antagonists or agonists, among others (3, 4). Furthermore, recent studies have uncovered that Eph and Ephrin can also engage in interactions with numerous non-Eph–Ephrin proteins (5), with the underlying mechanisms hypothesized to involve both *trans*-interactions between different cells and *cis*-interactions within the same cell (6). A study revealed that EFNB1/2 can bind to IL7R in a *cis*-interaction manner in T-lymphoma cells (7), serving as an example of the *cis*-acting mechanism. These findings underscore the intricate nature of Ephrin signaling system and highlight the potential for developing novel therapeutic approaches that target these alternative modes of activation.

N-glycosylation, a crucial post-translational modification for membrane proteins, plays a pivotal role in their function. A recent study highlights that N-glycosylation of PD-L1 significantly impacts the diagnostic accuracy and therapeutic efficacy of PD-L1–targeted therapies (8). The N-glycosylation of ephrin A1 (EFNA1) has been proven to be crucial for its function as a ligand to EphA2 (9). Similarly, glycoengineering of EphA4-Fc has been shown to enhance its stability and half-life, potentially improving its therapeutic potential and applications (10). However, the biological roles of glycosylation modifications within the Eph–Ephrin system, particularly in the context of Eph-independent Ephrin activation, remain inadequately explored. Furthermore, the antitumor effects of Eph–Ephrin-Fc recombinant proteins and their impact on the tumor microenvironment are still poorly understood.

Ephrin B1 (EFNB1), one of the B-type Ephrin ligands, serves as a crucial marker for mature B lymphocytes (11, 12). Our previous studies have illuminated the role of EFNB1 in fostering the malignant progression of lymphomas (13), alongside its close association with drug response, cellular origin, and prognosis in B-cell lymphomas (14). In the present study, we uncovered that EFNB1 primarily exists in the form of N-glycosylation and evaluated the implications of the N-glycosylation on EFNB1 function. Moreover, we have designed and purified both glycosylated and nonglycosylated EFNB1-RBD-Fc recombinant proteins, subsequently examining their antitumor efficacy as well as their influence on the tumor microenvironment. Furthermore, we have evaluated the influence of both intrinsic EFNB1 levels within tumor cells and the tumor microenvironment on the antitumor effects of EFNB1-RBD-Fc. This work provides crucial insights into the role of N-glycosylation in modulating Ephrin function as well as informing the design of Fc fusion protein therapeutics targeting Ephrin as agonists or antagonists.

## Results

### EFNB1 primarily exists as N-glycosylated protein

**Figure 1:**
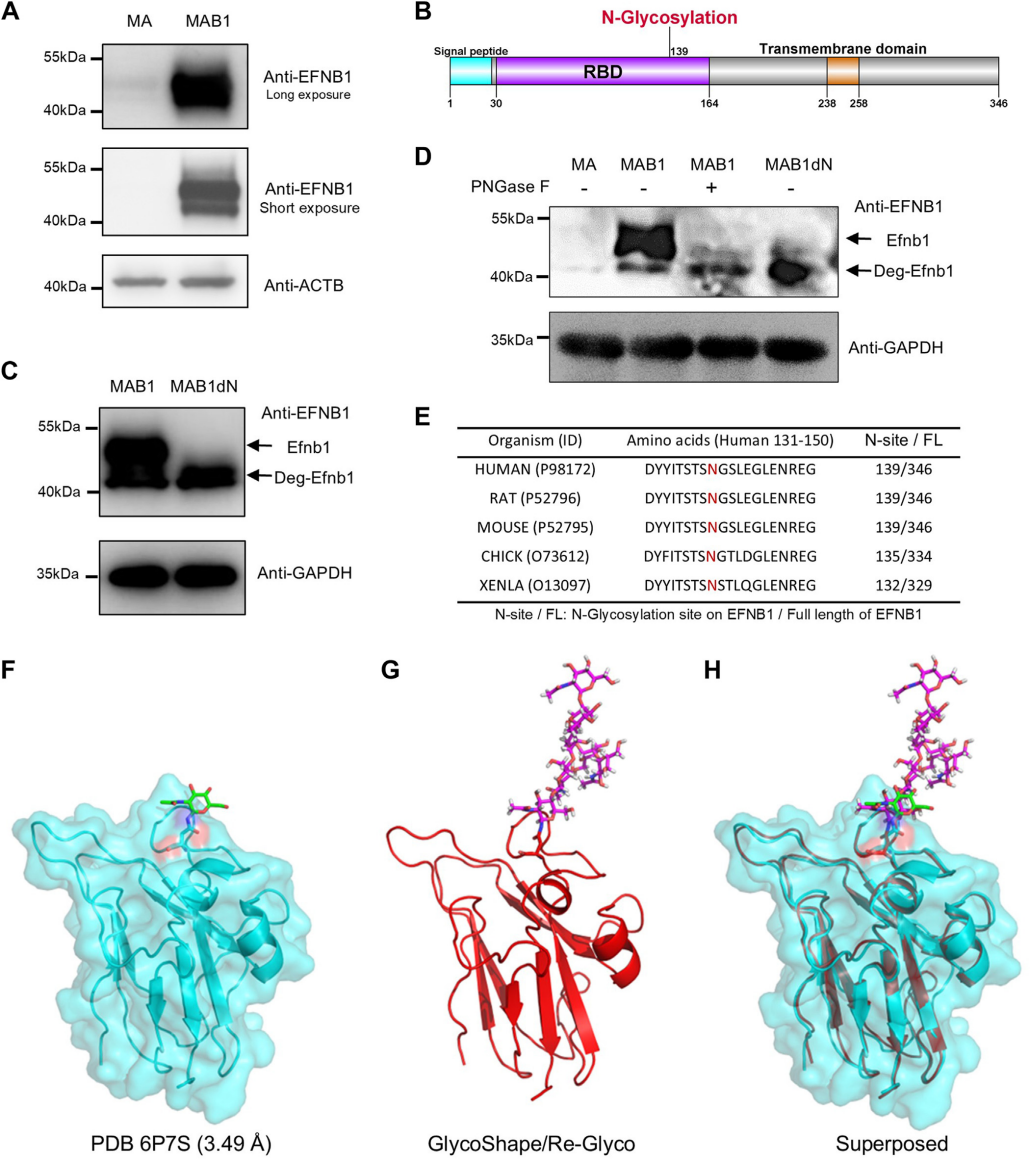
**N-glycosylation augments the molecular weight of EFNB1.***A*, Western blot analysis of EFNB1 bands in MA and MAB1. MAB1 refers to the stable cell line originated from MA cells, in which wildtype EFNB1 has been stably transfected and overexpressed. *B*, schematic representation of an N-linked glycosylation site within the RBD of EFNB1 protein across multiple species. The indicated features include the signal peptide, RBD, N-glycosylation site, and transmembrane domain. *C*, Western blot analysis of EFNB1 bands in MAB1 and MAB1dN. MAB1dN refers to the stable cell line originated from MA cells, in which the N-glycosylation-deficient EFNB1 has been stably transfected and overexpressed. *D*, Western blot analysis of EFNB1 bands in MAB1 with or without PNGase F treatment. MA and MAB1dN served as negative control in this experiment. *E*, the N-glycosylation sites of EFNB1 across various species. *F*, the crystal structure of the EFNB1-RBD (PDBe ID: 6P7S). EFNB1-RBD (PDBe ID: 6P7S) is represented in *cyan*. *G*, the predicted structure of the EFNB1-RBD with an N-glycan generated by the GlycoShape GDB and the Re-Glyco tool. The predicted structure of N-glycan within EFNB1-RBD is depicted in *magenta*, whereas the EFNB1-RBD itself is represented in *red*. *H*, the overlapping conformations of EFNB1-RBD with and without predicted N-glycan modifications. EFNB1, ephrin B1; PDBe, Protein Data Bank in Europe; RBD, receptor-binding domain.

Due to the presence of proteolytic cleavage sites within both the extracellular and intracellular domains of EFNB1, electrophoresis analyses frequently reveal the presence of bands with molecular weights lower than that of the full-length protein (15, 16). However, in the protein samples harvested from the stable cell line overexpressing EFNB1, named MAB1, we unexpectedly observed an additional band with a higher molecular weight and abundance, located in proximity to the expected band for the full-length EFNB1 protein (Figs. 1*A* and S1*A*).

We hypothesize that this larger band could represent a post-translational modification of EFNB1. To identify the modification responsible for the significant increase in the molecular weight of EFNB1, we first analyzed the post-translational modifications of EFNB1 and discovered the presence of a consensus sequence for N-glycosylation within its receptor-binding domain (RBD) (Fig. 1*B*). N139 is the only consensus sequence in EFNB1 for N-glycosylation. Given the substantial molecular weight contribution of N-linked glycosylation, this modification aligns with the observed increase in molecular weight. To verify if this potential N-glycosylation was responsible for the increased molecular weight of the EFNB1 protein band, we have mutated the 139th amino acid residue of EFNB1 from asparagine (N) to aspartic acid (D), resulting in the N-glycosylation-deficient N139D mutant. Furthermore, we have established a stable cell line overexpressing N139D mutant, named MAB1dN. Western blot analysis demonstrated the disappearance of the larger molecular weight band in MAB1dN (Figs. 1*C* and S1*B*). Furthermore, we conducted the PNGase F assay and found that the observed larger molecular weight band was lost in the PNGase F treatment (Figs. 1*D* and S1*C*), thereby confirming that N-glycosylation at N139 contributes to the elevated molecular weight of the EFNB1 protein band. In addition, we analyzed the conservation of this glycosylation site and found that it is highly conserved among multiple species (Fig. 1*E*), suggesting that this glycosylation modification serves an important functional role.

Glycosylation modifications play a crucial role in protein folding, stability, intercellular recognition, and interactions. However, the complex structures of glycan chains have always posed a significant challenge in the characterization of glycosylation. Recently, a study introduced a powerful tool, GlycoShape, along with the Re-Glyco algorithm (17). By employing the GlycoShape database and the Re-Glyco algorithm, we have predicted the N-glycan structure within EFNB1-RBD and compared it with its crystal structure with low modification of glycan (Fig. 1, *F*–*H*). Compared with the EFNB1-RBD with minor glycan chain modifications obtained through experiments (Fig. 1*F*), the GlycoShape results indicate that the EFNB1-RBD may possess high complex glycan chains (Fig. 1*G*). Notably, these large and complex glycan chains exert a relatively minor impact on the conformation of the EFNB1-RBD (Fig. 1*H*). In addition, we performed the CD analysis and observed that wildtype EFNB1-RBD-Fc with N-glycan modifications exhibits similar CD spectra to mutant EFNB1-RBD-Fc lacking N-glycan modifications (Fig. S2*A*).

### N-glycosylation is indispensable for the full functionality of EFNB1

**Figure 2:**
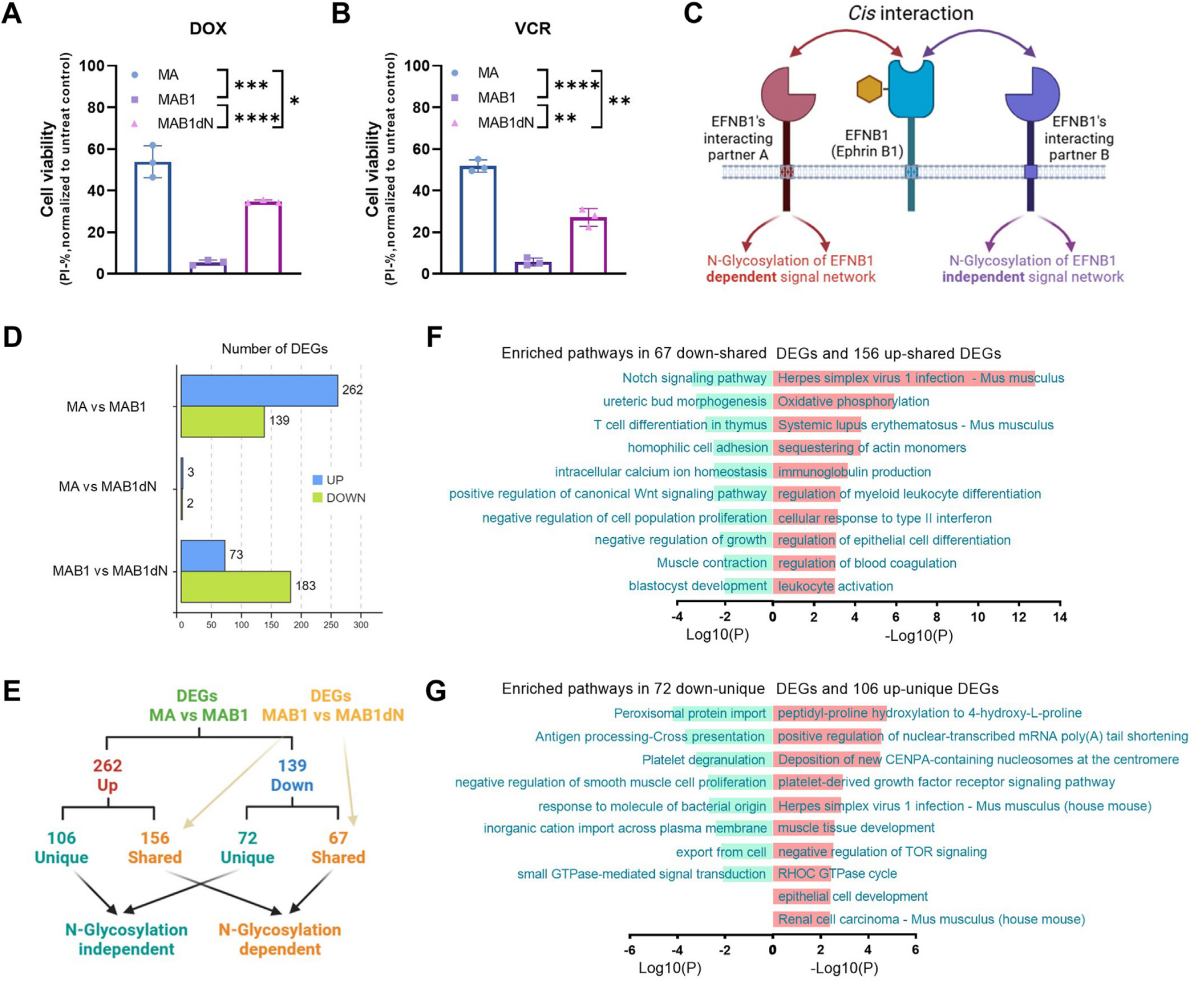
**N-glycosylation determines the transcriptional profile and drug sensitivity of EFNB1.***A*, cell viability following 48-h doxorubicin (DOX) treatment. Multiple unpaired *t* tests were used to test the significance. The dosage of DOX administered is 100 nM. *B*, cell viability following 48-h vincristine (VCR) treatment. The dosage of VCR administered is 15 nM. Multiple unpaired *t* tests were used to test the significance. *C*, the schematic diagram depicts both N-glycosylation-dependent and -independent *cis*-acting effects of EFNB1. Specifically, the effects mediated by interacting protein A are reliant on N-glycosylation modifications, whereas the effects of interacting protein B are independent of such modifications. *D*, the number of differentially expressed genes (DEGs) induced by wildtype EFNB1 and N139D mutant. The genes with the parameter of false discovery rate (FDR) below 0.05 and absolute fold change ≥2 were considered DEGs. *E*, DEGs of N-glycosylation-dependent and -independent downstream transcriptional network. *F* and *G*, enriched pathways of N-glycosylation-dependent (*F*) and N-glycosylation-independent (*G*) downstream transcriptional network. EFNB1, ephrin B1.

Previously, we had observed that overexpression of EFNB1 significantly enhanced the sensitivity of lymphoma cells to doxorubicin (DOX) and vincristine (VCR) (14). To investigate the impact of N-glycosylation on the biological functions of EFNB1, we conducted an analysis of the drug sensitivity profile of the N139D mutant. Notably, despite conferring sensitivity to drugs such as DOX and VCR in B-lymphoma cells, overexpression of the N139D mutant, deficient in N-glycosylation, resulted in a marked reduction in this sensitivity compared with wildtype EFNB1 (Fig. 2, *A* and *B*). These observations demonstrate the pivotal role that N-glycosylation plays in modulating the functions of EFNB1.

To assess the impact of N-glycan on protein–protein interactions, we analyzed the occupancy of N-glycan within the complex formed between EFNB1-RBD and its interacting proteins. We have retrieved the crystal structure depicting the interaction between the RBD of EFNB1 and a non-Eph receptor protein, specifically a viral attachment glycoprotein. Within this interaction, the N-glycosylation is localized to a disordered loop region, rather than at the interface where RBD interacts with glycoprotein (Fig. S2*B*). These results suggest that N-glycosylation may not directly impede the interaction between EFNB1-RBD and its interacting partners, and that the glycosylated and nonglycosylated forms of EFNB1 exhibit distinct biological effects (Fig. 2*C*).

Given the unique features of nonaggregated and suspended B-lymphoma cells, we propose a hypothesis that, unlike the classical Eph–Ephrin bidirectional signaling, EFNB1 expressed on these B-lymphoma cells may exert its effects predominantly through *cis*-interactions with its interacting proteins, where N-glycan potentially holds a crucial role in modulating the functionality of EFNB1. Identifying the interacting proteins of EFNB1 is crucial for understanding the functions of EFNB1 and its N-glycan modification. According to a recent study (18), EphA2, A4, B1, and B2 are potential Eph receptors for EFNB1 in B-lymphocytes. We analyzed the expression of the Eph receptors in the MA and MAB1 cells and found that only EphA2 was highly expressed (Fig. S2*C*), suggesting that EphA2 is the potential Eph interacting protein for EFNB1. Next, we utilized a mammalian recombinant protein expression system, employing the Expi293F, to produce murine EFNB1-RBD-Fc and EPHA2-RBD-His fusion proteins. However, we did not observe a direct interaction between Fc-tagged EFNB1-RBD and His-tagged EphA2-RBD in the pulldown assay (Fig. S2*D*). Given that we utilized nonaggregated and suspended B-lymphoma cells, which lack direct physical interactions between cells, it is not surprising that we failed to obtain the classical Eph–Ephrin bidirectional signaling interactions. Identifying the interacting proteins of EFNB1, as well as elucidating the roles of N-glycan within these interactions, continues to pose ongoing challenges. Although we have not yet identified the interacting proteins of EFNB1, based on our phenotypic data, we have been able to gain insights into the specificity and complexity of the *cis*-interaction mode.

To evaluate the N-glycosylation-dependent and -independent effects of EFNB1, we performed transcriptome sequencing on stable cell lines MAB1 expressing wildtype EFNB1 and MAB1dN expressing the N139D mutant variant. Differential gene expression (DEG) analysis revealed that compared with the parental MA cell line, MAB1 cells exhibited upregulation of 262 DEGs and downregulation of 139 DEGs, whereas MAB1dN cells displayed minimal alterations with only three upregulated and two downregulated DEGs (Fig. 2*D*). This stark contrast underscores the indispensable role of N-glycosylation in EFNB1-mediated transcriptional regulation. In addition, a comparative analysis between MAB1 and MAB1dN cells identified 73 upregulated and 183 downregulated DEGs in MAB1dN relative to MAB1 (Fig. 2*D*).

To discern the specific effects, we categorized the DEGs into two distinct sets: those altered in both MA *versus* MAB1 and MAB1 *versus* MAB1dN, designated as N-glycosylation-dependent downstream transcriptional network, and those exclusively altered in MA *versus* MAB1 but not in MAB1 *versus* MAB1dN, labeled as N-glycosylation-independent downstream transcriptional network (Fig. 2*E*). Subsequently, we conducted pathway enrichment analyses for both N-glycosylation-dependent and -independent downstream gene sets. Notably, within the N-glycosylation-dependent gene set, the most significantly impacted pathway was "herpes simplex virus 1 infection" (Fig. 2*F*). This observation suggests that EFNB1 overexpression mimics the activation of EFNB1 signaling induced by the interaction with this virus, a process reliant on N-glycosylation. In addition, other pathways influenced by N-glycosylation modifications encompassed oxidative phosphorylation and immune cell function–related signaling (Fig. 2*F*). Conversely, the enriched pathways within the N-glycosylation-independent downstream gene set displayed a less focused functional landscape (Fig. 2*G*).

In summary, our findings demonstrate that N-glycosylation modification is indispensable for the full functionality of EFNB1.

### N-glycosylation modulates the antitumor effects of EFNB1-RBD-Fc in the coculture system

**Figure 3:**
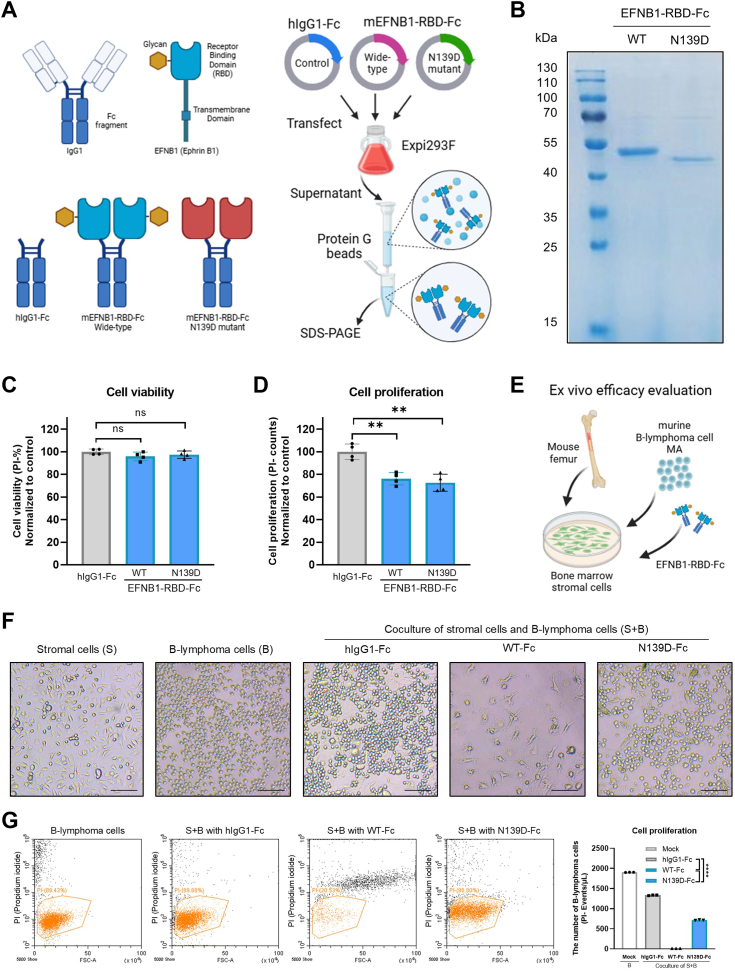
**The effects of EFNB1-RBD-Fc recombinant protein on stromal cells is contingent upon N-glycosylation.***A*, the schematic design of the EFNB1-RBD-Fc fusion protein, along with the process for its recombinant expression and purification. The EFNB1-RBD-Fc fusion protein is a genetically engineered construct that combines the RBD of EFNB1 with the Fc region of an immunoglobulin (typically IgG1). *B*, expression analysis of wildtype and N139D mutant EFNB1-RBD-Fc recombinant proteins. *C*, cell viability of lymphoma cells following treatment with EFNB1-RBD-Fc recombinant proteins. Multiple unpaired *t* tests were used to test the significance. *D*, number of lymphoma cells following treatment with EFNB1-RBD-Fc recombinant proteins. Multiple unpaired *t* tests were used to test the significance. *E*, coculture system of bone marrow stromal cells and B-lymphoma cells. After isolation, mouse bone marrow stromal cells were propagated through passages until they fully covered the bottom of the culture dish. Subsequently, MA B-lymphoma cells were seeded into the culture, with simultaneous addition of the EFNB1-RBD-Fc recombinant protein. *F*, representative images of bone marrow stromal cells, B-lymphoma cells, and their coculture. Photographs were taken after 48 h of coculture. The bar represents 40 μm. *G*, the effect of EFNB1-RBD-Fc on cell viability and proliferation of B-lymphoma cells. The PI-negative cell population is defined as the viable cell population. The PI-negative ratio is defined as cell viability. Multiple unpaired *t* tests were used to test the significance. EFNB1, ephrin B1; PI, propidium iodide; RBD, receptor-binding domain.

Given the significance of N-glycosylation in modulating EFNB1 activity, its potential impact on the antitumor efficacy of biopharmaceuticals, such as Fc fusion proteins, antagonistic peptides, and antibodies, holds great importance for guiding and optimizing the development of Eph–Ephrin RBD-based anticancer drugs or therapies. To this end, we utilized the Expi293F mammalian recombinant protein expression system to produce murine EFNB1-RBD-Fc fusion proteins (Fig. 3*A*). Protein electrophoresis confirmed that the molecular weights of both wildtype and N139D mutant EFNB1-RBD-Fc recombinant proteins were in accordance with expectations (Fig. 3*B*). Subsequently, we analyzed the effects of these EFNB1-RBD-Fc recombinant proteins on the cell viability and proliferation of MA B-lymphoma cells. Notably, both wildtype and mutant EFNB1-RBD-Fc fusion proteins exhibited partial inhibition of MA B-lymphoma cell proliferation with comparable inhibitory effects (Fig. 3, *C* and *D*), suggesting that the recombinant protein EFNB1-RBD-Fc possesses antitumor activity, manifested by its ability to inhibit cellular proliferation. Notably, this antitumor activity is independent of N-glycosylation modifications.

Given the pivotal role of Ephrin in intercellular signaling, we have devised an *ex vivo* coculture system involving bone marrow stromal cells and B-lymphoma cells to evaluate the interventional efficacy of EFNB1-RBD-Fc (Fig. 3*E*). Within this coculture paradigm, featuring stromal cells (S) and B-lymphoma cells (B), wildtype EFNB1-RBD-Fc exhibited a notable inhibitory effect on cellular proliferation (Fig. 3*F*).

Meanwhile, we also conducted a propidium iodide (PI) staining analysis on the B-lymphoma cells within the coculture system. We define the PI-negative cells as viable cells and the proportion of PI-negative cells as cell viability. In the coculture system supplemented with WT-Fc, we observed an abnormal cell population exhibiting unusually large forward scatter values (Fig. 3*G*), indicative of an enlarged cell size that is consistent with the characteristics of stromal cells. This suggests that a crucial effect of WT-Fc is to act on stromal cells and promote their detachment. Intriguingly, while the N139D-Fc also demonstrated a notable suppression of cell proliferation, it does not affect stromal cells (Fig. 3, *F* and *G*).

Collectively, these findings underscore the antitumor activity of EFNB1-RBD-Fc, highlighting the intricate nature of its antitumor mechanisms. Importantly, the antitumor efficacy of EFNB1-RBD-Fc is markedly influenced by the tumor microenvironment. Notably, our experiments suggest that N-glycosylation modification, though having limited impact on EFNB1-RBD-Fc's antitumor effect in a monocellular context, significantly modulates its efficacy within the coculture system.

### The endogenous EFNB1 levels in tumor cells affect the effect of EFNB1-RBD-Fc

Since the interaction of EFNB1 with binding partners may activate phosphorylation pathways, assessing the phosphorylation levels of downstream proteins can serve as an indicator of their signaling activation. However, quantitatively analyzing the downstream proteins of EFNB1 pose many technical challenges, and Western blot technique has a relatively limited range for detecting variations in protein levels. Alternatively, expression analysis of EFNB1’s downstream genes offers an optimal quantitative approach to determine whether EFNB1 signaling is activated or inhibited.

**Figure 4:**
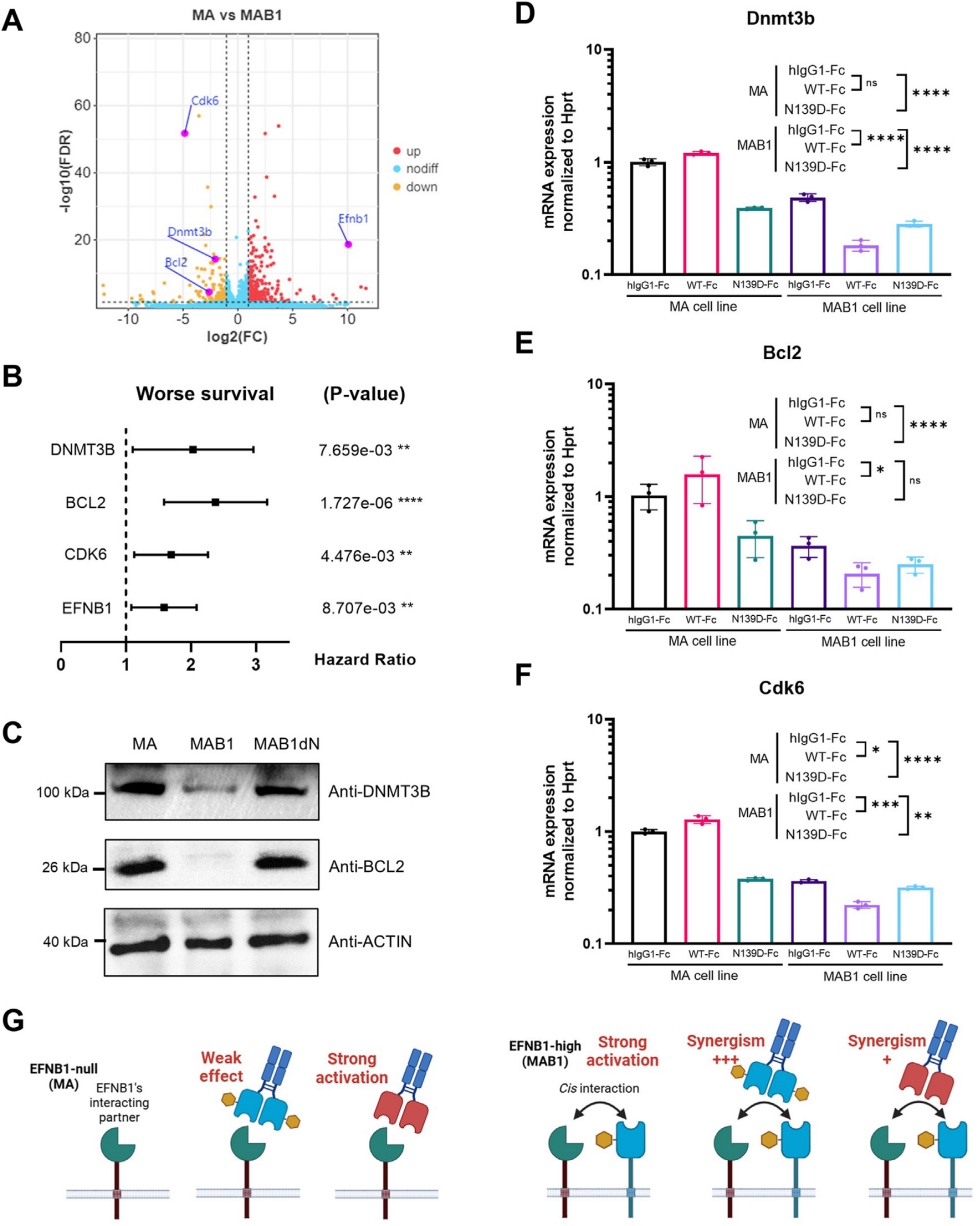
**The effect of EFNB1-RBD-Fc recombinant protein is modulated by both its N-glycosylation status and EFNB1 expression levels in tumor cells.***A*, volcano plot of differentially expressed genes (DEGs) in EFNB1-overexpressing cells. The genes with the parameter of false discovery rate (FDR) below 0.05 and absolute fold change ≥2 were considered DEGs. *B*, forest plot for the prognostic performance of CDK6, BCL2, and DNMT3B. *p* Value of the log-rank test were shown. A human diffuse large B-cell lymphoma dataset (Lenz Staudt Lymphoma GSE10846, n = 414) was chosen to survival analysis. The hazard ratio (HR), confidence interval, and *p* value in forest plot were obtained from the SurvExpress program. *C*, Western blot analysis of DNMT3B and BCL2 in MA, MAB1, and MAB1dN. *D*–*F*, the expression levels of Dnmt3b (*D*), Bcl2 (*E*), and Cdk6 (*F*) in Efnb1-null and EFNB1-high lymphoma cells following 24 h treatment with EFNB1-RBD-Fc recombinant protein. Multiple unpaired *t* tests were used to test the significance. *G*, hypothetical model of the intervention effects of EFNB1-RBD-Fc recombinant protein on both EFNB1-null and EFNB1-high cells. EFNB1, ephrin B1; RBD, receptor-binding domain.

To evaluate the impact of endogenous EFNB1 levels in tumor cells on the antitumor effects of EFNB1-RBD-Fc, we compared the influence of EFNB1-RBD-Fc on MA cells devoid of EFNB1 and MAB1 cells overexpressing EFNB1. Initially, we identified clinically relevant DEGs among MA and MAB1, Cdk6, Bcl2, Dnmt3b, as indicators of EFNB1 signaling (Fig. 4*A*). Prognostic analysis reveals that the expression levels of CDK6, BCL2, and DNMT3B are closely associated with the prognosis in human diffuse large B-cell lymphoma (Fig. 4*B*). The Western blot analysis revealed a significant downregulation of protein levels for both DNMT3B and BCL2 in the MAB1 cells with EFNB1 overexpression (Fig. 4*C*), which is in concordance with the transcriptome data. In addition, we made an attempt to blot for CDK6, but the outcome was unsuccessful (data not shown).

Subsequently, we treated MA and MAB1 cells using the EFNB1-RBD-Fc recombinant protein and examined the expression patterns of Cdk6-Bcl2-Dnmt3b genes. Our findings revealed distinct effects of EFNB1-RBD-Fc in MA and MAB1 cells. Specifically, WT-Fc solely upregulated the Cdk6 gene in MA cells, whereas it significantly downregulated Cdk6, Bcl2, and Dnmt3b genes in MAB1 cells (Fig. 4, *D*–*F*). In contrast, N139D-Fc markedly downregulated Cdk6, Bcl2, and Dnmt3b genes in MA cells and also significantly downregulated Cdk6 and Dnmt3b genes in MAB1 cells (Fig. 4, *D*–*F*). These results underscore the profound influence of EFNB1 expression levels in tumor cells on the regulatory effects of EFNB1-RBD-Fc on key genes, highlighting the complexity and diversity of EFNB1-RBD-Fc's mechanisms of action.

Although we have not yet identified the interacting proteins for EFNB1, based on the unique nonaggregating and suspension properties of lymphoma cells, along with the available experimental data, we have been able to gain insights into the specificity and complexity of the *cis*-interaction mode. (1) Since the B-lymphoma cells are suspended and nonaggregating, this excludes *trans*-interactions similar to those occurring between adherent cells or the culture dish or extracellular matrix. (2) No interaction was observed between EFNB1-RBD and EPHA2-RBD, and the remaining Eph receptors were found to be unexpressed. These findings suggest that EFNB1 may engage in *cis*-interactions with non-Eph proteins. (3) EFNB1-RBD-Fc can inhibit the proliferation of MA cells that lack EFNB1, suggesting that it recognizes potential non-Eph interactors on the cell surface, rather than functioning through competition with endogenous EFNB1. (4) Wildtype EFNB1-RBD-Fc influences the proliferation or apoptosis of stromal/B-lymphoma cells in coculture, indicating distinct non-Eph interactors on stromal cells compared with B-lymphoma cells.

Based on these insights, we propose the effects of EFNB1-RBD-Fc on the nonaggregated and suspended B-lymphoma cell as follows: The endogenous EFNB1 and the engineered EFNB1-RBD-Fc both exhibited transcriptional repression effects on all three target genes, suggesting that, within the context of this study, EFNB1-RBD-Fc should be characterized as an agonist rather than an antagonist. In EFNB1-null cells, the wildtype EFNB1-RBD-Fc displays weak effects, whereas the N139D mutant demonstrates robust effect on transcriptional repression. In contrast to EFNB1-null cells, EFNB1-high cells already reside in a state of robust effect on transcriptional repression conferred by EFNB1. Both wildtype and N-glycosylation-deficient EFNB1-RBD-Fc elicit synergistic effects, albeit with the N139D mutant displaying a weaker synergistic response (Fig. 4*G*). Given the limited research on Ephrin's non-Eph-dependent mechanisms, our data and the proposed model provide valuable insights for future studies.

## Discussion

The Eph–Ephrin signaling pathway holds significant importance in cancer, and targeting this pathway represents a potential antitumor therapeutic strategy. The B-lymphoma cells we employed in this study are nonaggregated suspension cells, lacking direct physical interactions between cells. Therefore, the traditional *trans*-interaction mode of Eph–Ephrin cannot be applied to B-lymphoma cell model. Overexpression of EFNB1, rather than a mutant lacking N-glycans, is sufficient to influence cellular biological functions such as drug sensitivity, suggesting that EFNB1 and its N-glycosylation possess significant biological effects, potentially functioning through *cis*-interactions. In this study, we have characterized the N-glycosylation modification of EFNB1 and delved into the potential effects of both EFNB1 and EFNB1-RBD-Fc, offering insights into the functionality of EFNB1 and facilitating drug development efforts targeting this molecule.

Identifying the *cis*-interacting proteins for EFNB1 and characterizing the interactions through N-glycans still remain ongoing challenges. A recent study (5) has identified numerous non-Eph–Ephrin receptors, such as EVC2, AQPEP, DSP4, for EFNB1 utilizing highly sensitive interaction assays, but the employed cell models exhibit limited relevance, thus casting doubt on their reproducibility in disease-specific contexts. Similarly, another study (18) pinpointed potential Ephrin receptors for EFNB1 in immune cells, including EphB1, EphB2, EphA2, EphA4, yet these discoveries too await validation in disease-relevant models. Although we conducted the pulldown assay to dig the interaction between EFNB1 and potential ligand, EphA2, results cannot give us a convincing conclusion that EFNB1 and EphA2 have direct interaction. Exploring the non-Eph *cis*-interacting proteins of EFNB1 under physiological conditions, including those within lymphoma cells themselves and stromal cells, will be an important research topic in the future.

The scope of this study leans toward applied research, with one of its highlights being the speculation on patterns of action for EFNB1 and EFNB1-RBD-Fc. This insight is significantly facilitated by our utilization of nonaggregating suspension cells, which simplifies the experimental process compared with the commonly employed adherent cells. The effects of EFNB1-RBD-Fc treatment on transcriptional repression in EFNB1-null MA cells mirrored those observed in EFNB1-overexpressing MAB1 cells, reinforcing our hypothesis that EFNB1-RBD-Fc directly engages with EFNB1's interacting partners. Considering the agonist potential of Eph–Ephrin-Fc proteins, rather than solely their antagonist role, offers crucial insights into future research and development of Eph–Ephrin-Fc therapeutics. Furthermore, Eph–Ephrin-Fc assumes a competitive stance with the endogenous Eph–Ephrin in tumor cells while simultaneously engaging non-Eph–Ephrin interacting proteins, fostering a synergistic effect. This dual mode of action underscores the complexity and potential of Eph–Ephrin-Fc molecules in modulating cellular processes and opens avenues for innovative therapeutic strategies.

N-glycosylation is a prevalent modification type of membrane proteins, playing a pivotal role in cell–cell and cell–extracellular matrix interactions. The Eph–Ephrin signaling pathway serves as a crucial conduit for signal transduction between adjacent cells. The Eph–Ephrin signaling pathway is one of the essential pathways regulating the interactions and signaling between adjacent cells. Notably, a study has shown that glycosylation significantly enhances the binding and activation of EphA2 by EFNA1 (9). Conversely, the deglycosylated form of EFNA1 exhibited markedly reduced affinity for EphA2, emphasizing the indispensable role of glycosylation in modulating Eph–Ephrin interaction. Another study has demonstrated that Ephrin ligands with distinct conformations and combinations are capable of eliciting distinct Eph signaling responses (19). In our study, we found that the consensus sequence for N-glycosylation is located within a disordered region, exerting minimal influence on the conformation of the EFNB1-RBD. Furthermore, this N-glycan does not reside at the interaction interface between EFNB1-RBD and the viral glycoprotein. Consequently, we hypothesize that the N-glycan on EFNB1-RBD possess high plasticity, potentially affecting interactions with *cis*-interacting proteins. This hypothesis necessitates further interrogation by future structural biology studies.

Collectively, this study has elucidated the functional roles of N-glycosylation within EFNB1, including its influence on drug response and the contrasting effects of glycosylated *versus* nonglycosylated forms on the transcriptome and signaling pathways. In addition, we also determined the intervention of EFNB1-RBD-Fc on B-cell proliferation and the stromal cells. These findings suggests that N-glycosylation of EFNB1 possess significant biological functions and influence the therapeutic potential of Fc proteins. Furthermore, we anticipate that our study will ignite interest among drug developers in recognizing the broader significance of glycosylation modifications for optimizing and evaluating the therapeutic efficacy of protein-based drugs, emphasizing that glycosylation serves a multifaceted purpose beyond merely stabilizing proteins and extending their half-lives. Our findings underscore the complexity and versatility of the Eph–Ephrin system, prompting a re-evaluation of traditional agonist or antagonist design strategies and opening new avenues for innovative therapeutic interventions aimed at modulating tumor progression.

## Experimental procedures

### Cell line and constructs

The MA cell line, derived from the *Eμ-Myc;Cdkn2a*^*−/−*^ cell line and recently established in our laboratory (20), is maintained in a specialized B-cell medium (BCM) comprising of 45% Dulbecco's modified Eagle's medium, 45% Iscove's modified Dulbecco's medium, and 10% fetal bovine serum. The BCM was further supplemented with 100 U/ml of penicillin and streptomycin as well as 25 μM β-mercaptoethanol. The authenticity of the cell line was validated using RNA-Seq. In addition, a routine PCR-based screening for mycoplasma contamination is performed every 2 weeks.

The complementary DNA (cDNA) of EFNB1 was cloned into the retroviral vector MLS (LTR-cDNA-SV40-GFP) in previous study (13), whereas the N139D mutant plasmid was generated through a PCR-based mutagenesis approach in this study. In addition, the Fc fusion protein expression plasmid was obtained by synthetically constructing the EFNB1-RBD fragment and subsequently inserting it into an Fc plasmid backbone in pcDNA3.4 vector. The murine wildype and N139D-mutant EFNB1-RBD (Ala25–Ala170) were each cloned into a modified pcDNA3.4 vector, which featured an IL-2 signal sequence at the N terminus and a hIgG1 Fc at the C terminus. Similarly, the murine EPHA2-RBD (Ala25–Ala170) was cloned into a modified pcDNA3.4 vector, which featured an IL-2 signal sequence at the N terminus and a His tag at the C terminus.

### Establishment of stable cell lines

To generate stable cell lines, retroviral plasmids were cotransfected with helper plasmids into 293 cells using calcium phosphate–mediated transfection, resulting in the production of viral particles. These viral particles were then used to infect MA cells, followed by fluorescence-activated cell sorting to isolate GFP-positive cells, indicating successful integration and expression of the transgene.

### Protein extraction and Western blot analysis

MA cells cultured in suspension were collected by centrifugation and lysed using radioimmunoprecipitation assay buffer on ice. After the addition of loading buffer, the samples were heated at 95 °C for 10 min and stored at −20 °C for later use. For Western blot analysis, 7.5% SDS-PAGE gels were employed to separate proteins, followed by standard blotting and detection procedures. Rabbit polyclonal anti-EFNB1-RBD (HPA067188; Sigma, 1:1000 dilution), rabbit polyclonal anti-DNMT3B (A2899; Abclonal, 1:1000 dilution), and rabbit polyclonal anti-Bcl2 (A19693; Abclonal, 1:1000 dilution) were employed for the study. Rabbit monoclonal anti-ACTB (AC026; Abclonal, 1:100,000 dilution) and mouse monoclonal anti-GAPDH (AC002; ABclonal, 1:10,000 dilution) were used as internal control. The specificity of each antibody used has been validated by the company through methods such as Western blot.

### Deglycosylation of cell lysates

Cells were lysed in radioimmunoprecipitation assay buffer (150 mM NaCl, 50 mM Tris [pH 7.5], 1% Nonidet P-40, and protease inhibitor mixture). Following lysis, the cell lysates were denatured through heating at 100 °C for 10 min and then rapidly chilled on ice. Twenty microliters of the denatured samples was incubated at 37 °C overnight, either without or with the addition of 2 μl of PNGase F, a purified enzyme obtained from our laboratory. After this incubation period, the samples were subjected to Western blot analysis.

### CD analysis

CD spectroscopy is a rapid, simple, and relatively accurate method for assessing protein secondary structure in solution. The purified recombinant EFNB1-RBD-Fc protein, at a concentration of 0.1 mg/ml in 0.05x PBS buffer, was utilized for CD analysis using a JASCO J-815 spectrometer.

### Transcriptome sequencing and analysis

Suspension-cultured MA cells were collected by centrifugation and lysed in Trizol reagent (15596018; Invitrogen) on ice, followed by storage at −80 °C. RNA was extracted according to the Trizol kit's protocol and shipped on dry ice to Gene Denovo Biotechnology Co for transcriptome sequencing.

The differential expression analysis of RNAs was performed by DESeq2 software between two different groups. The genes with the parameter of false discovery rate below 0.05 and absolute fold change ≥2 were considered DEGs.

Pathway enrichment analysis of DEGs was performed with Metascape (https://metascape.org). Terms with a *p* value <0.01, a minimum count of 3, and an enrichment factor >1.5 (the enrichment factor is the ratio between the observed counts and the counts expected by chance) are collected and grouped into clusters based on their membership similarities. More specifically, *p* values are calculated based on the accumulative hypergeometric distribution.

### Drug sensitivity assay

The drug concentrations were set by lethal dosage tests. Cell viability and lethal dosage (LD20–LD60) at 48 h post-treatment, measured by PI negativity, were used for drug response analysis across most cell strains. After 24 h, the media were replenished with an equal volume of fresh media. At 48 h post-treatment, cells were stained with PI and subjected to flow cytometry analysis. PI-negative cells were considered viable, and the proportion of this population was used as a measure of cellular viability. DOX (S1208) and VCR (S1241) were purchased from Selleck.

### Recombinant protein expression and purification

Wildtype and N139D-mutant EFNB1-RBD-Fc, along with EphA2-RBD-His, were transiently expressed in Expi293F cells. Expi293F cells were cultured in OPM-293 CD05 medium (OPM Biosciences Co, Ltd) at 37 °C supplemented with 5% CO_2_ in a ZCZY-CS8 shaker (Shanghai Zhichu Instrument Co, Ltd) at 120 rpm. When the cell density reached 2.5 × 10^6^ to 3.0 × 10^6^ cells per mL, the cells were transiently transfected with the expression plasmids. Approximately 1.5 mg of plasmids was premixed with 4.5 mg of polyethylenimines (Polysciences) in 50 ml of fresh medium for 30 min before application. For transfection, 50 ml of the mixture was added to 1 l of cell culture, and the transfected cells were harvested 96 h after transfection.

Cells were collected by double centrifugation, and Fc-tagged proteins in the supernatant were purified by HiTrap Protein G affinity column (Qiagen), whereas His-tagged proteins were purified by Ni–NTA affinity column (Qiagen). The eluate was then concentrated and purified by high-resolution gel filtration column Superdex 200 Increase 10/300 (GE HealthCare). Peak fractions of the targets were confirmed by SDS-PAGE followed by Coomassie blue staining.

### Fc-tagged protein pull-down

Ten micrograms of purified wildtype and N139D-mutant EFNB1-RBD-Fc was each incubated with 2 μg of EphA2-RBD-His for 2 h at 4 °C. Following incubation, the Fc-tagged EFNB1 mixed with His-tagged EphA2 were loaded onto a Protein G column. Flowthroughs were collected, and subsequently, eluates were eluted using citric acid at a pH of 3.0 and also collected. Both the flowthroughs and eluates were then loaded onto SDS-PAGE for analysis.

### Treatment with Fc recombinant proteins

In the experiments designed to investigate cellular proliferation effects, Fc recombinant proteins were coincubated with cells for 48 h, followed by PI staining and subsequent flow cytometry analysis. The proportion of PI-negative viable cells was defined as cellular vitality, whereas their density was recorded as a measure of cell count. Across all experiments, a dosage of 10 μg/ml was uniformly administered for both hIgG1-Fc and EFNB1-RBD-Fc.

### Establishment of primary bone marrow stromal cells

After isolating the femur bones from 6- to 8-week-old female mice, the bone marrow stromal cells were flushed out using a syringe. Following red blood cell lysis, filtration, and centrifugation, the cells were resuspended in BCM and plated in culture dishes for static cultivation. Once the cells adhered to the surface, nonadherent cells were removed. Upon reaching confluence, the stromal cells were passaged at a ratio of 1:2, with one-third of the original culture medium being retained during the passage process.

### Prognostic analysis

Survival analysis was conducted utilizing the online SurvExpress tool (21), where a human diffuse large B-cell lymphoma dataset (Lenz Staudt Lymphoma GSE10846 (22), n = 414) was selected for analysis. The prognostic index was derived by the expression value and employed a Cox model to stratify patients into distinct risk groups. The risk grouping was further optimized using an integrated algorithm within the SurvExpress program, adhering strictly to the provided tutorial for accurate execution.

### Gene expression analysis

MA cells either lacking EFNB1 expression or overexpressing EFNB1 (designated as MAB1 cells) were treated with Fc recombinant protein for 8 h. Following treatment, RNA was extracted, reverse-transcribed, and subjected to quantitative PCR (qPCR) analysis.

Total RNA was extracted using Trizol reagent kit (15596018; Invitrogen) according to the manufacturer’s protocol. cDNA was produced by reverse transcription of total RNA using HiScript III 1st Strand cDNA Synthesis Kit (R312; Vazyme) according to the manufacturer’s protocol. Quantitative real-time PCR was performed with AceQ SYBR Green I qPCR master mix (Q511; Vazyme) using QuantStudio three System (Thermo), according to the manufacturer’s protocol. Hprt was used as a reference gene. The ΔΔCt method was used to calculate relative expression levels. All primers used in qPCR were listed as following.

mHprt—Forward, 5′-TCAGTCAACGGGGGACATAAA-3′

mHprt—Reverse, 5′-GGGGCTGTACTGCTTAACCAG-3′

mCdk6—Forward, 5′-TGCTCAACCCATCGAGAAGT-3′

mCdk6—Reverse, 5′-GCTGGATTAAACGTCAGGCA-3′

mBcl2—Forward, 5′-GAGTACCTGAACCGGCATCT-3′

mBcl2—Reverse, 5′-ATCAAACAGAGGTCGCATGC-3′

mDnmt3b—Forward, 5′-CCAAGCGCCTCAAGACAAAT-3′

mDnmt3b—Reverse, 5′-TGACTTCAGAAGCCATCCGT-3′

### Statistical analysis

All the experiments were performed in biological and technical triplicates. Values reported in figures are expressed as the SD of the mean, unless otherwise indicated. Data were tested using multiple unpaired *t* tests, log-rank test as indicated in figure legends. *p* Values of <0.05 were considered significant. ∗*p* < 0.05, ∗∗*p* < 0.01, ∗∗∗*p* < 0.001, and ∗∗∗∗*p* < 0.0001. Statistical analyses were performed with GraphPad Prism 9.0 (GraphPad Software, Inc).

## Ethics approval

All animal experiments were approved by the Animal Care and Use Committee of Laboratory Animal Research Center, Jiangsu University.

## Data availability

All raw data generated or analyzed throughout this study are accessible upon reasonable request from the corresponding author. RNA-Seq data pertaining to this investigation have been deposited in the National Center for Biotechnology Information Sequence Read Archive under the following accession code: PRJNA1104361. Any cell strains and constructs generated in this study can be acquired through the Materials Transfer Agreement.

## Supporting information

This article contains supporting information.

## Conflict of interest

The authors declare that they have no conflicts of interest with the contents of this article. The authors declare that the research was conducted in the absence of any commercial or financial relationships that could be construed as a potential conflict of interest.
