# Supplementary material for: N-glycosylation of ephrin B1 modulates its function and confers therapeutic potential in B-cell lymphoma
Source: J Biol Chem. 2025 Jan 27;301(3):108229. doi: 10.1016/j.jbc.2025.108229 (PMC11871495; doi:10.1016/j.jbc.2025.108229)
Supplement: Supporting information [file mmc1.pdf]

Supporting Information

N-glycosylation of ephrin B1 modulates its function and confers therapeutic potential in B-cell lymphoma

Xiaoxi Li *et al.*

Correspondence to: Xiaoxi Li, lixiaoxi@ujs.edu.cn; Hua Tang, tanghua@nju.edu.cn

List of Supporting Information

Fig. S1. Original uncropped western blot images.

Fig. S2. The impact of N-Glycan on EFNB1-RBD and its potential Eph interactions.

Supplementary Figure 1

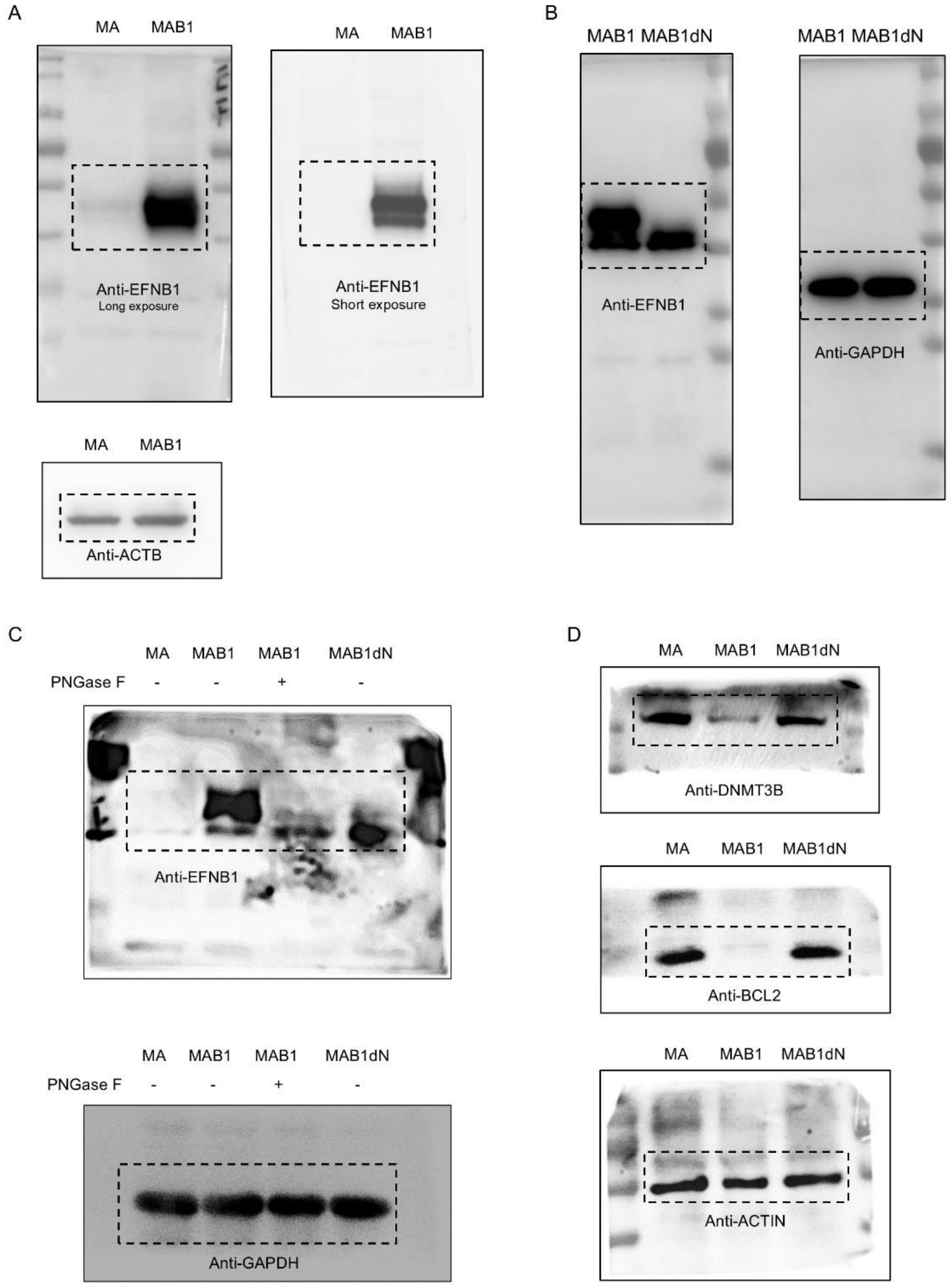

Supplementary Figure 1. Original uncropped western blot images. A. The uncropped western blot image corresponding to Fig. 1A. B. The uncropped western blot image corresponding to Fig. 1C. C. The uncropped western blot image corresponding to Fig. 1D. D. The uncropped western blot image corresponding to Fig. 4C.

Supplementary Figure 2

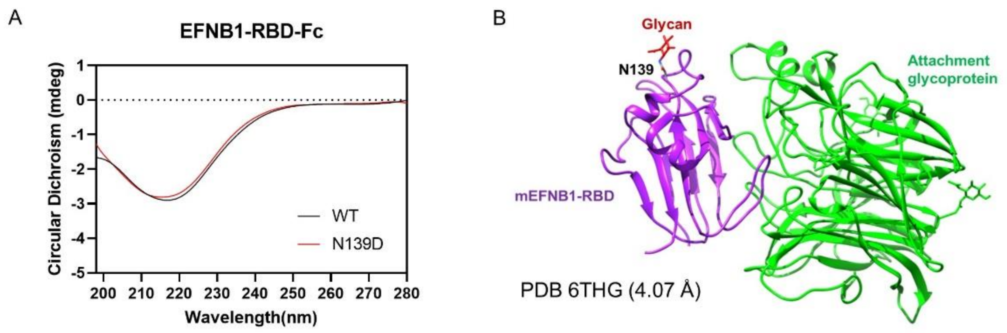

C

Expression level (FPKM)		
Eph receptor	MA	MAB1
Epha1	0.001	0.001
Epha2	4.12	2.415
Epha3	0.001	0.001
Epha4	0.001	0.001
Epha5	0.001	0.001
Epha6	0.001	0.001
Epha7	0.001	0.001
Epha8	0.005	0.001
Epha10	0.001	0.005
Ephb1	0.001	0.001
Ephb2	0.001	0.001
Ephb3	0.001	0.001
Ephb4	0.1	0.045
Ephb6	0.001	0.001

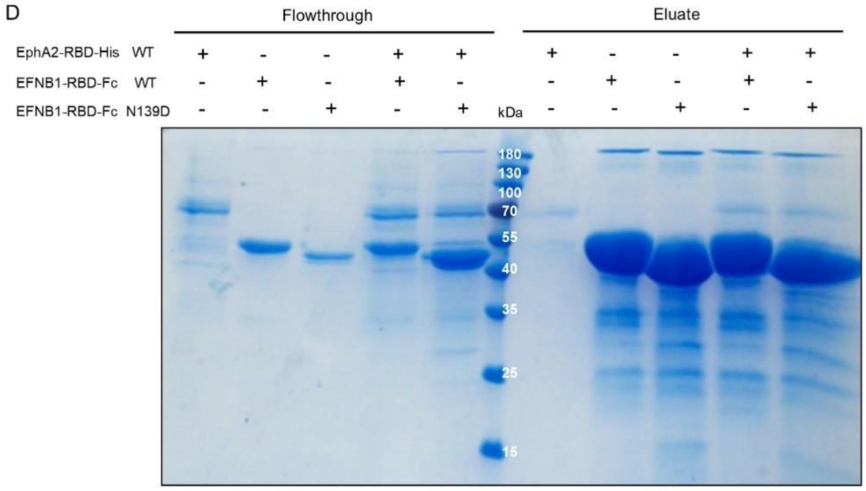

Supplementary Figure 2. The impact of N-Glycan on EFNB1-RBD and its potential Eph interactions. A. The circular dichroism spectra of wild-type EFNB1-RBD-Fc with N-glycan and Mutant EFNB1-RBD-Fc (N139D). B. The crystal structure of the EFNB1-RBD in complex with viral attachment glycoprotein globular domain (PDBe ID, 6P7S). EFNB1 is represented in purple. The N-glycosylation sites of EFNB1 is represented in red. Viral glycoprotein is represented in green. C. The expression levels of Eph receptors in MA and MAB1. D. The Fc-tagged protein pull down assay to analysis the interaction between EFNB1-RBD and EphA2-RBD.
